# Integration of Evidence on Community Cancer Risks from Elongate Mineral Particles in Silver Bay, Minnesota

**DOI:** 10.1111/risa.13673

**Published:** 2021-02-02

**Authors:** Linda D. Dell, Alexa E. Gallagher, Lisa J. Yost, Kenneth A. Mundt

**Affiliations:** ^1^ Ramboll Amherst MA USA; ^2^ Ramboll Minneapolis MN USA; ^3^ Cardno Chemrisk Boston MA USA

**Keywords:** Ambient air monitoring, community cancer risks, elongate mineral particles

## Abstract

The potential for cancer‐related risks to community members from ambient exposure to elongate mineral particles (EMPs) in taconite processing has not been formally evaluated. We evaluated 926 ambient air samples including 12,928 EMPs (particle structures with length‐to‐width ratio ≥3:1) collected over 26 years near a taconite processing facility in Silver Bay, Minnesota. Eighty‐two percent of EMPs were ≤3 μm in length and 97% of EMPs had an average aspect ratio <20:1. A total of 935 (7.3%) EMPs had length >5 μm and AR ≥3:1. Average ambient concentration of NIOSH countable amphibole EMPs over all years was 0.000387 EMPs per cubic centimeter (EMP/cm^3^). Of 12,765 nonchrysotile EMPs, the number of amphiboles with length and width dimensions that correlate best with asbestos‐related carcinogenicity ranged from four (0.03%) to 13 (0.1%) and the associated ambient amphibole air concentrations ranged from 0.000003 to 0.000007 EMP/cm^3^. After 65 years of taconite processing in Silver Bay, evidence of an increased risk of mesothelioma and lung cancer in community members who did not work in the taconite industry is lacking. The absence of an increased risk of asbestos‐related cancer in the Silver Bay community is coherent with supporting evidence from epidemiological and toxicological studies, as well as ambient exposure data and lake sediment data collected in Minnesota Iron Range communities. Collectively, the data provide consistent evidence that nonasbestiform amphibole minerals lack the carcinogenic potential exhibited by amphibole asbestos.

## 1 INTRODUCTION

**Fig 1:**
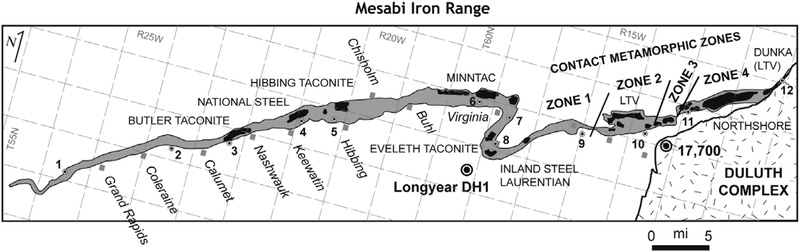
Simplified geologic map of the Mesabi Iron Range. Adapted from McSwiggen and Morey (2008, Fig. 1).

The Mesabi Range is comprised of iron‐bearing rock and slate that runs along an east‐west axis for about 120 miles in northeastern Minnesota. Iron ore—hematite, and more recently, taconite—has been mined continuously since the early 1890s. Geologists have identified four distinct geological zones ranging from an unmetamorphosed iron formation in the west (zone 1) to a highly metamorphosed iron formation in the east (zone 4), the latter the result of magma intrusions from the Duluth Complex more than a billion years ago (Fig. 1) (Jirsa, Miller, & Morey, 2008; McSwiggen & Morey, 2008). Consequently, minerals found in the Mesabi Iron Range (MIR) are heterogeneous across the range and include phyllosilicates (minnesotatite, greenalite, and stilpnomelane) found on the western end of the range and amphiboles (hornblende, cummingtonite‐grunerite, and ferroactinolite) found on the eastern end of the range (Jirsa et al., 2008; McSwiggen & Morey, 2008).

Small communities dot the Mesabi Range. At the western end of the range, the communities where taconite is (or was) mined and milled include Keewatin, Hibbing, Chisholm, Mountain Iron, Virginia, Eveleth, Gilbert, Biwabik (all located in zone 1), and Aurora and Hoyt Lakes (located in or adjacent to zone 2). Taconite at the eastern end of the range has been mined in Babbitt (located in zone 4) and the ore transported via rail for processing in Silver Bay, Minnesota, approximately 50 miles south of the range, since 1955.

In the early 1970s, the U.S. Environmental Protection Agency (EPA) filed suit against Reserve Mining Company, the owner and operator of the Silver Bay taconite processing facility, because of its practice of disposing taconite ore tailings in Lake Superior. Elongate mineral particles (EMPs) initially characterized as “asbestos‐like” amphibole fibers had been found in the Duluth water supply (Cook, Glass, & Tucker, 1974). The “asbestos‐like fibers” were identified as cummingtonite‐grunerite minerals from the taconite tailings.

Following a series of litigation rulings, Reserve Mining ceased disposal of tailings into Lake Superior and constructed an on‐land tailings basin. A 1975 court order directed that the Silver Bay processing facility reduce the fiber count in the ambient air at Silver Bay “below a medically significant level,” a level which was not defined (Reserve Mining Company vs. the US EPA, 1975). In the absence of clear health risks associated with the ingestion and inhalation of these EMPs, the Court ordered that the facility comply with a requirement that emission “controls may be deemed adequate which will reduce the fiber count to the level ordinarily found in the ambient air of a control city such as St. Paul.” This requirement became known as the “control city standard.” Since the requirement was established, the Silver Bay processing facility has routinely collected and submitted ambient air samples for microscopic analysis according to protocols established by the Minnesota Department of Health (MDH) and the Minnesota Pollution Control Agency (MPCA). MDH Method 852 defines a fiber for counting as a “parallel‐sided mineral particle having an aspect ratio of at least 3:1 and may or may not exhibit diffraction contrast” using transmission electron microscopy (TEM) (MDH, 1991a). This definition of “fiber” also is consistent with the definition of EMP (any mineral particle with a minimum aspect ratio of 3:1) (National Institute of Occupational Safety and Health [NIOSH], 2011). This definition of “fiber” or EMP should not be confused, however, with definitions of “fiber” in various asbestos regulations, which specify counting certain mineral particles with lengths >5 μm and length‐to‐width (or aspect) ratios of ≥3:1 or ≥5:1. EMPs include asbestiform mineral fibers and nonasbestiform particles of minerals, such as cleavage fragments generated during comminution processes. We direct the reader to Bailey, Chatfield, Gibbs, and Wylie (2018) for an overview of the “universe of particles.”

The Occupational Safety and Health Administration (OSHA) and Mining Safety and Health Administration (MSHA) specify methods for analysis and counting of fibers, primarily using phase‐contrast microscopy (PCM), in their asbestos regulations. The PCM method, however, does not discriminate between asbestos and other mineral particles, but identifies EMPs that meet criteria for asbestos fibers based on morphology. TEM analysis is often used following PCM to assist with mineral identification, and to potentially discriminate populations of cleavage fragments from populations of asbestos fibers (Van Orden, Allison, & Lee, 2008). OSHA and MSHA exclude cleavage fragments and nonasbestiform minerals from asbestos regulations, while the National Institute of Occupational Safety and Health (NIOSH) Recommended Exposure Limit (REL) does not. For the REL, NIOSH (2011) defined a “countable EMP” as mineral particles meeting the length >5 μm and aspect ratio (AR) ≥3:1 using phase contrast microscopy (PCM) as specified in NIOSH Analytical Method 7400 (“A” Rules).

Early epidemiological studies of taconite workers did not report increased risks of mesothelioma or lung cancer or pulmonary changes consistent with asbestosis (Clark, Harrington, Asta, Morgan, & Sargent, 1980; Cooper, Wong, & Graebner, 1988; Cooper, Wong, Trent, & Harris, 1992; Higgins, Glassman, Oh, & Cornell, 1983). Higgins et al. (1983) did not find mesothelioma reported on death certificates among workers at Reserve Mining while Cooper et al. (1988, 1992) reported one death from mesothelioma in former workers at the Erie and Minntac mines. The Erie mine, now closed, is located in zone 2 with some pits located in zone 3 [S. Gischia, personal communication, August 3, 2020] while the Minntac mine is located in zone 1. In Norway, autopsy examinations of lung tissue in two former taconite miners who died from lung cancer also found pulmonary fibrosis in one decedent (smoking history unknown) and silicosis in the other decedent, described as a heavy smoker (Gylseth, Norseth, & Skaug, 1981). These miners had worked for 27 years and 36 years in the mine, respectively. Mesothelioma was not reported (Gylseth et al., 1981).

During the years 1988–2006, cancer surveillance conducted by the Minnesota Department of Health found men in Northeast Minnesota experienced a two‐fold excess of mesothelioma (146 cases vs. 69 expected based on the Minnesota average), an excess suggestive of occupational exposure to amphibole asbestos (MDH, 1999, 2003a, 2003b, 2007). Cases of mesothelioma in taconite workers, identified by the MDH starting in 1997, raised questions again about the potential risks associated with exposure to the amphibole EMPs encountered in processing the taconite ore (Brunner, Williams, & Bender, 2008; MDH, 1997, 1999, 2003a, 2003b, 2015). Brunner et al. (2008) and the MDH (2003b) reported some of these cases included a history of occupational exposure to commercial asbestos in jobs inside and outside the mining industry, and these exposures likely explained the excess mesothelioma risks.

Previously, Gamble and Gibbs (2008) reviewed toxicology and epidemiology evidence related to exposure to amphibole cleavage fragments and concluded that risks of mesothelioma and lung cancer were not likely associated with exposure to amphiboles in nonasbestiform mineralogical habits. Addison and McConnell (2008) reviewed carcinogenicity studies of asbestiform and nonasbestiform tremolite and concluded shorter, thicker fibers were less hazardous than thinner, longer fibers of the same mineral. In addition to tremolite studies, these reviews considered studies of lung tumors and pleural tumors in male rats exposed via intratracheal instillation to fibrous ferroactinolite (Coffin, Palekar, & Cook, 1982; Cook, Palekar, & Coffin, 1982). The fibrous ferroactinolite was collected from “loose surface iron‐formation rocks” in the eastern mine. Cook et al. (1982) described *in vivo* longitudinal splitting of the ferroactinolite fibers, a characteristic of asbestiform material.

Since these reviews were published, Cyphert et al. (2012, 2016) compared the toxicity of ferroactinolite cleavage fragments collected from an iron ore mine in Ontario (ON), Libby amphibole (LA) asbestos, El Dorado (ED) tremolite, and Sumas Mountain (SM) chrysotile. In these studies, ON, LA, ED, and SM samples were prepared using elutriation to generate particles that were respirable sizes for rats (aerodynamic diameter < 2.5 um). The ON sample was described as a nearly pure sample of ferroactinolite cleavage fragments, with mean length of 1.1 μm (± 0.9) comprised of 14% fibers (AR≥5:1). Cyphert et al. (2012) reported doses of 0.5 mg and 1.5 mg ON delivered via intratracheal instillation induced acute inflammation one day after exposure. After 15 months, however, no fibrosis or tumors were observed in any of the rats exposed to the ON cleavage fragments, unlike rats exposed to LA, ED, and SM minerals (Cyphert et al., 2016).

Cook et al. (2016) conducted a statistical analysis of dose data for 10 different mineral or synthetic fiber types characterized according to length, width, shape, particle number, and thickness. Cook et al. (2016) evaluated the doses and the fiber characterization according to parameters such as sample leaching, sum of elongate particles, sum of surface area, and sum of elongate particle mass in mathematical models to optimize theoretical fits between fiber type and mesothelioma potency. Some of the samples used in the analysis were acid leached to simulate changes that would occur in the animal (*in vivo*). Cook et al. (2016) reported that EMP surface area, and not width or length dimensions, provided the optimum dose–response model regardless of the composition of elongate particles.

Previously, Wilson, McConnell, Ross, Axten, and Nolan (2008) used ambient air monitoring concentrations of EMPs at the Northshore facility to calculate health risks to the Silver Bay community. Wilson et al. (2008) used a conservative assumption that the nonasbestos EMPs were as potent as amosite (grunerite asbestos), and calculated health risks of approximately 35 excess lung cancers and mesotheliomas combined per one million lifetimes. Under an alternative assumption that the nonasbestos EMPs were as potent as chrysotile, the health risks were negligible (fewer than 0.77 excess asbestos‐related lung cancers and mesotheliomas combined per one million lifetimes). Wilson et al. (2008) reported the mean airborne concentration of EMPs >5μm in Silver Bay was less than 0.00014 EMP/cm^3^, based on nine samples collected in 1998 and analyzed using transmission electron microscopy. This concentration is consistent with ambient concentrations of asbestos fibers in remote locations (<0.001 f/cm^3^, length >5 μm) (WHO, 1986) or median estimates of continuous asbestos exposure in the general population (0.0004 f/cm^3^, length >5 μm) (NRC, 1984).

The objective of this article is to consider potential mesothelioma and lung cancer risks to the Silver Bay community in relation to potential ambient exposures to EMPs emitted as part of taconite ore processing operations. We consider more than 20 years of ambient air monitoring data collected by the current operators of the Northshore facility in the Silver Bay community according to features and size criteria proposed by other researchers that (1) help explain the cancer potency of asbestos or (2) discriminate populations of nonasbestiform EMPs, including cleavage fragments, from populations of asbestos. We integrate existing evidence regarding potential hazards of amphibole and nonamphibole EMPs encountered in taconite mining and milling by workers to inform potential cancer risks for residents of Silver Bay from exposure to ambient EMPs.

## 2 DATA SOURCES AND METHODS

Operators of the Northshore facility have collected ambient air monitoring data for over 28 years. The ambient air samples were collected and analyzed according MDH Method 852 (MDH, 1991a) to calculate fibers per cubic meter (fibers/m^3^) in air. The ambient air monitoring data were collected as single 96‐hour samples every 12th day from two locations near Silver Bay. The two sampling locations were selected by state regulators to evaluate potential particle emissions from the Silver Bay taconite processing plant (station 1) and from the tailings disposal basin (station 2), approximately five miles inland from Lake Superior. MDH Method 852 uses an indirect sample preparation and specifies transmission electron microscopy (TEM) to count particles from samples collected on Millipore methyl cellulose ester filters with a 0.8 micrometer pore size. All particles with parallel sides and an AR ≥3:1 were counted. MDH Method 852 states that that it “cannot distinguish between asbestiform fibers and cleavage fragments of the same mineral on a fiber by fiber basis” but the particle size distributions of the sample can be used to place the sample as consistent with cleavage fragments or consistent with asbestiform fibers. MDH Method 852 also specifies energy dispersive X‐ray (EDX) analysis to allow for characterization of each structure on chemical composition based on diffraction patterns. The EMPs were categorized broadly into the following four categories: amphibole, chrysotile, not amphibole and not chrysotile, or ambiguous (i.e., not easily recognized as amphibole, chrysotile, or nonamphibole). Specific chemistry was available for a subset of the amphibole structures to distinguish amphibole minerals (cummingtonite‐grunerite, hornblende, actinolite, tremolite, and “other”).

We conducted a descriptive analysis of the existing concentration data (EMPs per cubic meters [EMP/m^3^]) from 1990 to May 2018. The dataset is inclusive of nearly 13,000 individual structures. Data for individual structures (length, width, primary mineralogy, chemistry) were available for a subset of the years, January 1996–December 2004 and January 2006–May 2018. Individual structure data were not available for 2005, and this is believed to be the year when the state laboratory first contracted with a private laboratory to conduct the EMP analyses (S. Gischia, personal communication, March 12, 2019).

We used the Northshore dataset to calculate EMP concentration data according to several size‐based fractions which have been hypothesized to explain differences in asbestos‐related potency for mesothelioma and/or lung cancers by different investigators. Stanton, Miller, May, Tegeris, and Morgan (1981) proposed amphibole EMPs with length >8 μm and width ≤0.25 μm are the most potent for asbestos‐related carcinogenicity. Lippmann (1988, 2014) suggested that asbestos amphibole EMPs that are most potent for mesothelioma have length >5 μm and width <0.1 μm while asbestos EMPs that are most potent for lung cancer have length >20 μm and width <0.4 μm. Berman and Crump (2003) proposed an “optimal index” for predicting all health‐related effects based on asbestos EMPs of length >10 μm and width <0.4 μm.

Other counting criteria have been proposed for distinguishing between asbestiform fibers and cleavage fragments or other nonasbestiform EMPs. OSHA (1992) described cleavage fragments formed during the comminution of minerals as typically showing AR <20:1. As a simplified approach, Chatfield (2010, 2018) proposed identifying potentially asbestiform EMPs using TEM and counting EMPs with AR ≥20:1 and width <1.5 μm. The American Society for Testing and Materials (ASTM) developed the *Standard Practice for Sampling and Counting Airborne Fibers, including Asbestos Fibers, in Mines and Quarries, by Phase Contrast Microscopy and Transmission Electron Microscopy* (D7200‐06) to identify and discriminate countable EMPs (length >5 μm using NIOSH 7400 “A” rules) that are likely to be asbestiform from EMPs of similar dimensions that are likely to be nonasbestiform cleavage fragments or prismatic crystals. Harper, Lee, Doorn, and Hammond (2008) used ASTM D7200‐06 to evaluate the nonasbestiform amphibole EMPs encountered in taconite mining and processing on the eastern end of the MIR. This standard used PCM initially to identify particles that exhibit features that are common to asbestos fibers (that is, show curvature, split ends, or a bundle of fibrils). Such particles are assigned to Class 1, and are considered potentially asbestiform, regardless of dimensions. Particles with parallel sides and length >10 μm *or* width <1 μm are assigned to Class 2 and are also assumed to be potentially asbestiform. Separately, Harper et al. (2008) evaluated an alternative definition for Class 2 particles (length >10 μm *and* width <1 μm), which had been proposed by the committee responsible for developing the ASTM D7200 standard. Class 3 particles are identified as all other particles that meet the NIOSH definition of EMP when observed under PCM, including possible cleavage fragments. In other words, Class 1 and 2 are considered potentially asbestiform and require further evaluation by TEM. Interlaboratory testing of ASTM D7200‐06, however, reported poor agreement between laboratories regarding consistent identification of characteristics and features common to asbestos, despite demonstrating consistency in the measurement of the dimensions of the particles (Harper, Lee, Slaven, & Bartley, 2012). ASTM 7200–06 was withdrawn and replaced with ASTM 7200–12. In the revised standard, “countable fibers” (length >5 μm, aspect ratio >3:1) are categorized as follows: Group 1 are fibers with width <1.0 μm; Group 2 are fibers with width >1.0 μm, but which show curvature or have splayed ends, or have the appearance of a bundle; and Group 3 are all other countable fibers. Groups 1 and 2 are considered potentially asbestiform and require additional evaluation using TEM.

After categorizing the EMP data according to the different size‐based fractions, we calculated the rolling (or moving) annual average of ambient EMP concentrations of nonasbestiform amphibole EMPs with lengths >5 μm using the formula:

$$\frac{y_{1}+y_{2}+\cdots +y_{m}}{m}\;;\;\frac{y_{2}+y_{3}+\cdots +y_{m+1}}{m}$$

where *y*_1_ *, y*_2_ *, …* are the values of concentration data representing a one‐year moving average *m*.

This method eliminates short‐term fluctuations and smooths the trend lines. We plotted the rolling annual average with the concentration data in figures.

We calculated an exposure concentration limit for the community using the NIOSH recommended exposure limit (REL) for countable EMPs (length >5 μm, AR ≥3:1) of 0.1 EMP/cm^3^ (averaged over 100 minutes), a value derived to protect health over a 45‐year working life assuming 240 days of occupational exposure per year. We extrapolated from this occupational exposure to a continuous ambient exposure for community members using the following approach:

$$EC\;=\frac{\left(CA\;\times \;ET\;\times \;EF\;\times \;ED\right)}{AT}\;$$

Where,

EC is exposure concentration limit, CA (EMP/cm^3^) is concentration in air, ET (h/day) = exposure time, EF (days/year) = exposure frequency, ED (years) is exposure duration, and AT (lifetime in years x 365 days/year x 24 h/day) = averaging time

Using this equation, we used the NIOSH REL of 0.1 EMP/cm^3^ averaged over 100 minutes (equivalent to 1.67 h/day) for 240 days/year for a 45‐year working life and calculated an equivalent exposure concentration of 0.0029 EMP/cm^3^ (applied to NIOSH countable EMPs > 5 μm, AR ≥ 3:1) for a resident assuming continuous exposure over a 70‐year lifetime.

$$\begin{array}{ccc} \mathrm{EC} & & =\;0.1\frac{\mathrm{EMP}}{{\mathrm{cm}}^{3}}\;\times \frac{1.67}{24}\mathrm{hours}\;\times \;\frac{240}{365}\mathrm{days}\times \\ & & \;\frac{45}{70}\mathrm{years}=0.0029\;\frac{\mathrm{EMP}}{{\mathrm{cm}}^{3}} \end{array}$$

## 3 RESULTS

### 3.1 Description of EMP Mineralogy

The Northshore dataset included a total of 12,928 EMPs (AR≥3:1) in 926 samples from 1996–2018: 6,175 (48%) showed an electron diffraction pattern consistent with amphibole minerals; 5,673 (44%) showed diffraction patterns that were not amphibole or not chrysotile; 917 (7%) did not give clear diffraction patterns (or were obscured by other minerals) and were classified as “ambiguous”; 160 (1%) showed a diffraction pattern consistent with chrysotile; and three were missing information to classify in one of these four categories. EMPs with a diffraction pattern consistent with chrysotile or missing chemistry information were excluded from further analyses. Cummingtonite‐grunerite and actinolite comprised 69% and 30% of the 2,569 amphibole EMPs with chemical composition, respectively. Tremolite and hornblende each comprised less than 1% of the amphibole EMPs. When restricted to 935 EMPs with length >5 μm (i.e., a NIOSH countable EMP), 629 (67%) showed a diffraction pattern consistent with amphiboles.

### 3.2 EMP Dimensions and Concentrations

Only 7.3% of 12,765 total EMPs counted according to MDH 852 were longer than 5 μm with AR ≥3:1, while 10.2% of 6,175 amphibole EMPs counted according MDH 852 were longer than 5 μm with AR ≥3:1. Only 48 (0.8%) of amphibole EMPs had length >5 μm, width <1.5 μm, and AR ≥20:1, dimensions that are commonly reported in populations of amphibole asbestos. The average ambient air concentration of NIOSH countable amphibole EMPs was 0.000387 EMP/cm^3^ (Table I). Few of the counted amphibole EMPs met the length and width criteria associated with mesothelioma or lung cancer potency described by Lippmann (2014), Stanton et al. (1981), and Berman and Crump (2003): 4, 4, and 13 amphibole EMPs, respectively. When counting only these EMPs, ambient air concentrations ranged from 1.5 × 10^−6^ to 4 × 10^−7^ EMP/cm^3^ (Table I).

**Table I:** Sample Concentrations of EMPs Sized According to the MDH Method 852, NIOSH 7400, ASTM D7200 and Other Size Descriptions, 1996–2018 (*n* = 926)*

		Total EMP (Excluding Chrysotile)			EMP (Amphibole Only)		
Reference/Method	Counting criteria	No. EMPs Counted/ Meeting Criteria (%)	No. Samples with ≥1 EMP Meeting Criteria	Mean Concentration (EMP/cm^3^)	No. Amphibole EMPs Counted/ Meeting Criteria	No. Samples with ≥1 Amphibole EMP Meeting Criteria	Mean Concentration (EMP/cm^3^)
*MDH Method 852* (uses TEM)	AR ≥3:1 ^†^	12,765^†^	926	0.0089197	6,175	847	0.004306
*NIOSH 7400* (uses PCM)							
Countable EMP^‡^	L >5, AR ≥3	935 (7.3)	491	0.000574	629	349	0.000387
*Chatfield (2010, 2018)*							
Potentially asbestiform or asbestos^‡^	L >5, W<1.5, AR >20:1	100 (0.7)	90	0.000064	48	44	0.000030
*ASTM D7200‐06*							
Class 2^‡^	L >10 or W <1, AR≥3:1	457 (3.6)	301	0.000276	318	218	0.000192
Alternative Class 2 ^‡^	L >10 and W <1, AR≥3:1	45 (0.4)	43	0.000029	26	25	0.000014
*ASTM D7200‐12*							
Group 1	L >5, W <1, AR ≥3:1	326 (2.6)	238	0.000202	218	164	0.000140
Group 2	L >5, W ≥1, AR ≥3:1	609 (4.8)	382	0.000371	411	267	0.000247
*Stanton et al*. (*1981*)							
Most carcinogenic fibers^§^	L >8, W ≤0.25	20 (0.3)	19	0.000011	10	10	0.000006
*Lippmann (2014)*							
Mesothelioma	L >5, W ≤0.1	17 (0.1)	17	0.000011	4	4	0.00
Lung cancer	L >20, W <0.4	6 (<0.1)	6	0.000004	4	4	0.000003
*Berman and Crump (2003)*							
Optimal exposure index	L >10, W <0.4	24 (0.2)	22	0.000015	13	12	0.000007
*OSHA* (*1992*)							
Cleavage fragment (not asbestiform)	AR ≤20:1	12,399 (97.1)	924	0.008636	6,075	847	0.004240
Cleavage fragment (not asbestiform)	L >5, AR ≤20:1	835 (6.5)	458	0.000509	581	334	0.000357

Abbreviations: AR, aspect ratio; EMP, elongate mineral particles; L, length in μm; PCM, phase contrast microscopy; TEM, transmission electron microscopy; W, width in μm.

* 926 samples containing information on 12,928 EMPs.

^†^ Of the 12,928 EMPs, 31 had an aspect ratio of <3:1, 160 (1.2%) were chrysotile, and 3 were missing information to classify chemistry. EMPs that were chrysotile or missing information to classify chemistry were excluded from additional analyses.

^‡^ Based on counting criteria using phase contrast microscopy, which cannot distinguish between asbestiform or nonasbestiform or amphibole or nonamphibole EMPs.

^§^ Based on asbestiform and nonasbestiform minerals.

Under the criteria for ASTM D7200‐06, 5,692 amphibole EMPs were included as “potentially asbestiform” based on length >10 μm *or* width <1 μm. However, only 26 amphibole EMPs had length >10 μm *and* width < 1 μm. The difference of 5,666 are EMPs with length >5 and ≤10 μm. Descriptive information regarding features of asbestos that would allow for classification under Class 1 “asbestiform” was not available, because this ASTM standard was not considered by the analysis laboratory; however, Harper et al. (2008) evaluated EMPs from a sample of crushed ore sourced from Northshore taconite facility and reported that nearly none of the EMPs had features consistent with the definition of Class 1, potentially asbestiform. Therefore, nonasbestiform amphibole cleavage fragments were likely to be misclassified as potentially asbestiform under ASTM D7200‐06 (Table I).

Under ASTM D7200‐12, the 629 countable amphibole EMPs were essentially divided into two groups: Group 1, comprised of 218 EMPs with width < 1 μm (median width, 0.7 μm, and median AR, 10.8:1) and Group 2, comprised of 411 EMPs with width ≥1 μm (median width, 1.5 μm, and median AR, 4.8) (Table II). The ASTM D7200‐12 groupings more closely match those proposed by Chatfield (2010, 2018) for discriminating potentially asbestiform minerals (length >5 μm, width <1.5 μm, AR ≥20:1) from likely cleavage fragments that meet countable EMP criteria (length >5 μm, and AR <20:1). In the former group, EMPs had median width of 0.3 μm and in the latter group, EMPs had median width of 1.3 μm (Table II).

**Table II:** Mean and Median Amphibole EMP Size Data for Length, Width and Aspect Ratio According to the TWHS (Hwang et al., 2013,2014), ASTM D7200 and Other Definitions, 1996–2018

		Length (μm)		Width (μm)		Aspect ratio (n:1)	
Reference/Method	Counting criteria	Mean (SD)	Median (range)	Mean (SD)	Median (range)	Mean(SD)	Median(range)
MDH Method 852^†^ (TEM)	AR≥3:1	2.2 (2.1)	1.5 (0.07, 40.0)	0.4 (0.4)	0.3 (0.01, 7.0)	6.9 (7.2)	5.0 (0.05, 280.1)
	EMPs, except chrysotile	2.2 (2.1)	1.5 (0.07, 40.0)	0.4 (0.4)	0.3 (0.01, 7.0)	6.8 (6.9)	5.0 (0.05, 280.1)
	Amphibole EMPs	2.5 (2.4)	1.7 (0.1, 40.0)	0.5 (0.5)	0.3 (0.03, 7.0)	6.4 (5.1)	5.0 (0.05, 105.0)
	NA/NC EMPs	1.9 (1.7)	1.4 (0.07, 35.0)	0.3 (0.3)	0.3 (0.01, 5.0)	7.1 (8.4)	5.0 (0.05, 280.1)
*Restricted to amphibole EMPs (n = 6,175)* *							
Potentially asbestiform							
*NIOSH 7400* (PCM)	L>5, AR≥3	8.1 (3.5)	7.0 (5.0, 40.0)	1.3 (0.8)	1.2 (0.1, 7.0)	9.3 (11.3)	5.8 (3.0, 105.0)
*Chatfield (2010, 2018)* (SEM/TEM)	L>5, 0.04<W<1.5, AR>20:1	9.6 (4.4)	8.7 (5.1, 23.0)	0.3 (0.1)	0.3 (0.1, 0.8)	41.1 (21.5)	34.5 (20.0, 105.0)
*ASTM D7200‐06*							
Class 2 criteria	L>10 or (W <1), AR≥3:1	9.3 (4.4)	8.0 (5.0, 40.0)	1.2 (1.0)	0.8 (0.1, 7.0)	13.7 (14.6)	9.4 (3.0, 105.0)
Alternative Class 2 criteria	L>10 and W<1, AR≥3:1	13.1 (4.0)	11.0 (10.0, 23.0)	0.5 (0.3)	0.4 (0.1, 0.9)	39.1 (27.0)	32.1 (11.9, 105.0)
*ASTM D7200‐12*							
Group 1	L>5, W<1, AR≥3:1	7.4 (2.8)	6.4 (5.0, 23.0)	0.6 (0.2)	0.7 (0.1, 0.9)	17.2 (16.4)	10.8 (6.1, 105.0)
Group 2	L>5, W≥1, AR≥3:1	8.5 (3.8)	7.0 (5.1, 40.0)	1.7 (0.7)	1.5 (1.0, 7.0)	5.1 (1.7)	4.8 (3.0, 13.8)
*Stanton et al*. (*1981*)							
Mesothelioma	W≤0.25, L>8	10.8 (4.4)	9.5 (8.6, 23.0)	0.2 (0.1)	0.2 (0.1, 0.3)	66.6 (24.0)	66.1 (36.0, 105.0)
*Lippmann (2014)*							
Mesothelioma	W≤0.1, L>5	7.8 (2.3)	7.4 (5.7, 10.5)	0.1 (0)	0.1 (0.1, 0.1)	77.5 (22.6)	74.0 (57.0, 105.0)
Lung cancer, asbestosis	W<0.4, L>20	21.3 (1.4)	21.1 (20.0, 23.0)	0.3 (0)	0.3 (0.3, 0.3)	76.9 (10.9)	73.3 (69.0, 92.0)
*Berman and Crump (2003)*							
Optimal exposure index	W<0.4, L>10	14.0 (5.2)	11.0 (10.0, 23.0)	0.3 (0.1)	0.3 (0.1, 0.3)	59.7 (23.5)	58.8 (31.3, 105.0)
*OSHO* (*1992*)							
Cleavage fragments	AR≤20:1	2.4 (2.3)	1.7 (0.1, 40.0)	0.5 (0.5)	0.3 (0.1, 7.0)	5.9 (2.8)	5.0 (0.05, 20.0)
Cleavage fragments	L>5, AR≤20:1	8.0 (3.4)	7.0 (5.0, 40.0)	1.4 (0.8)	1.3 (0.3, 7.0)	6.7 (3.3)	5.5 (3.0, 18.7)

* 926 samples containing information on 12,928 EMPs.

^†^ Includes 31 of 12,928 EMPs and 23 of 6,175 amphibole EMPs with aspect ratio of less than 3.

^‡^ Lippmann 2014 mesothelioma: width <0.1 μm and length <5 μm;  lung cancer and asbestosis: width <0.4 μm and length >20 μm.

^§^ TWHS = Taconite Workers Health Study, NIOSH = National Institute for Occupational Safety and Health, ATSM = American Society for Testing and Materials, EMP = elongate mineral particle, SD = standard deviation, μm = micrometer, aspect ratio = length/width, NA/NC = nonamphibole, nonchrysotile.

**Fig 2:**
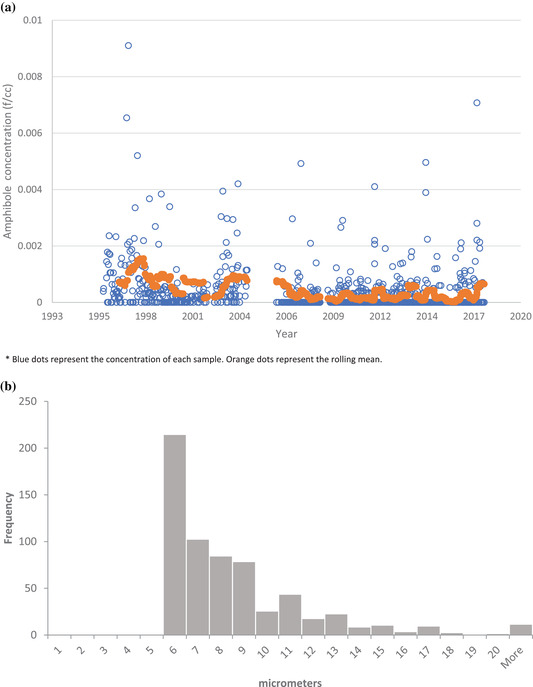
(a) Concentration of amphibole EMPs with lengths >5 μm, 1996–2018 (*n* = 926). (b) Distribution of lengths for amphibole EMPs with lengths >5 μm, 1996–2018 (*n* = 629).

**Fig 3:**
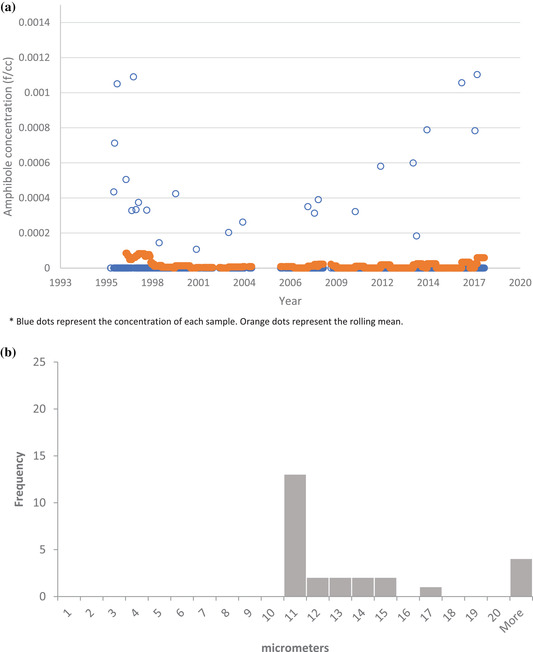
(a) Concentration of amphibole EMPs with length >10 μm and width <1 μm, 1996–2018 (*n* = 926). (b) Distribution of lengths for amphibole EMPs with lengths >10 μm and widths <1 μm, 1996–2018 (*n* = 26).

Overall, the size characteristics of the EMPs are consistent with a population of cleavage fragments: 6,075 (98%) of 6,175 of amphibole EMPs had aspect ratios ≤20:1). The rolling annual average ambient EMP concentrations of the population of amphibole EMPs with length >5 μm was calculated and plotted with the concentrations of each individual sample (Fig. 2a). In the 2006–2018 period, the rolling average concentrations ranged between 0.000016 EMP/cm^3^ and 0.00077 EMP/cm^3^. The distribution of the lengths of the amphibole EMPs with length >5 μm is provided in Fig. 2b; most of these EMPs fell in the 5–10 μm range. The rolling annual average ambient EMP concentrations of nonasbestiform amphibole EMPs with lengths >10 μm and widths <1 μm is noticeably lower (Fig. 3a). The length distribution of the few EMPs meeting the length >10 μm and width <1 μm criteria is provided in Fig. 3b. Supplemental materials (Figs. S1 and S2 in the Supporting Information provide similar figures for the concentration of amphibole EMPs without applying a length or width restriction criteria).

### 3.3 Comparison of EMP Concentrations to Exposure Concentration Limit Derived from NIOSH REL

For amphibole EMPs, we calculated an average concentration of 0.000387 EMP/cm^3^ for NIOSH countable EMPs (L >5 μm and AR ≥3:1), and even lower average concentrations for other size‐based descriptions (Table I). This value is approximately 7.5 times lower than the NIOSH REL after extrapolation from an occupational exposure scenario to a 70‐year lifetime continuous exposure scenario for residents (0.0029 EMP/cm^3^ of continuous exposure, see Methods). Furthermore, 0.000387 EMP/cm^3^ represented the average of 96‐hour samples collected every 12 days (or approximately 30 times per year). After allowing for this sampling duration and sampling frequency, the exposure concentration for continuous exposure for community members is 0.000129 EMP/cm^3^ (calculated as 0.000387 EMP/cm^3^ x [96 h/24 h] x [30.4 days/365 days]). Consequently, the potential exposure of community members to ambient amphibole EMPs with length >5 μm is more than a full order of magnitude below an exposure concentration limit allowing for continuous exposure over a 70‐year lifetime.

## 4 DISCUSSION

Epidemiological studies of community members are not available to evaluate cancer risks to the population from ambient exposure to EMPs related to taconite mining. Other evidence, however, is available to evaluate qualitatively whether community health risks are increased from ambient EMP exposures from taconite emissions. The evidence includes occupational epidemiology studies of taconite workers; cancer surveillance of Minnesota residents; toxicology studies of EMPs, especially nonasbestos EMPs or cleavage fragments; as well as ambient air monitoring data and lake sediment data from MIR communities.

### 4.1 Evidence from the Taconite Workers Health Study (TWHS)

Hwang, Ramachandran, Raynor, Alexander, and Mandel (2013, 2014) characterized exposures to EMPs in taconite mining and processing. Allen, Alexander, MacLehose, Ramachandran, and Mandel (2014) reported excess lung cancer mortality for taconite miners employed one year or more overall when compared to the Minnesota population (SMR 1.16, 95% CI 1.09–1.24) (Table III). Lung cancer mortality rates were elevated for each geological zone (Table III). Lung cancer mortality did not increase with duration of employment when comparisons were made to workers employed 2 to 5 years (Table III). After accounting for cigarette smoking using a probabilistic bias adjustment, the lung cancer SIR was attenuated (SIR 1.1, 95% CI 1.0–1.3, Table III) (Allen et al., 2015a). In a nested case‐control study of lung cancer among taconite workers, no increased risk of lung cancer was observed in relation to duration of employment in taconite mining, by department, or by cumulative exposure to EMPs (length >5 μm and an aspect ratio (AR) >3:1) per cubic centimeter per year (EMP/ cm^3^‐years) categorized according to quartiles, or EMP/ cm^3^‐years by geological zone (Zone 1, Zone 2, and Zone 4), after adjusting for silica exposure, previous employment in hematite mining, or exposure to commercial asbestos (Allen et al., 2015b) (Table IV).

**Table III:** Lung Cancer and Mesothelioma Risk Estimates in Cohort Studies of Taconite Workers

					Lung Cancer			Mesothelioma		
Author and Year	Study Population	Study Period	Comparison	Exposure Categories	No.Obs	Risk Estimate*	95% CI	No.Obs	Risk Estimate*	95% CI
Higgins et al. (1983)	5,751 taconite workers, Reserve Mining	1952−1976	MN mortality rates	Worked ≥1 year	15	0.84	0.47−1.38	0	NR	
				Worked ≥1 year & <15 years since hire	7	0.70	0.28−1.43			
				Worked ≥1 year & ≥15 years since hire	8	1.02	0.44−2.00			
Cooper et al. (1988, 1992)	3,444 taconite workers, Erie/LTV Steel & Minntac/US Steel Corp	1959−1988	MN mortality rates	Worked ≥3 months	62	0.87	0.52−0.86	1	NR	
Allen et al. (2014)	31,067 taconite mining workers at seven companies	1960−2010	MN mortality rates	Overall	949	1.16	1.09‐1.24	30	0.77	1.87−3.96
				*By geographic zone*						
				Zone 1	NR	1.18	1.09−1.27	NR	1.85	0.98−3.16
				Zone 2	NR	1.48	1.26−1.63	NR	7.38	4.3−11.82
				Zone 4	NR	1.23	1.07−1.40	NR	3.17	1.37−6.25
				*By employment duration*						
				1 year	123	1.10	0.81−1.26	4	1.14	0.34−3.81
				2–5 years	250	1.0	Referent	8	1.0	Referent
				6–14 years	239	1.01	0.85−1.21	6	0.77	0.26−2.25
				≥15 years	337	0.94	0.79−1.13	12	1.08	0.44−2.67
Allen et al. (2015a)	40,720 taconite mining workers	1988−2010	MN cancer incidence rates	Employed at one of Seven companies	973	1.1^†^	1.0−1.3	51	2.4	1.8−3.2

* SMR (Allen et al., 2014; Higgins et al., 1983, Cooper et al., 1992; Allen et al. 2014 (overall, by geographic zone); SRR (Allen et al., 2014, (by employment duration); SIR (Allen et al., 2015a).

^†^ SIR adjusted for smoking.

Abbreviations: MN, Minnesota; mppcf, million particles per cubic foot; NR, not reported; SIR, standardized incidence ratio; SMR, standardized mortality ratio; SRR, standardized rate ratio; yr, year.

**Table IV:** Case‐Control Studies of Lung Cancer and Mesothelioma Among Taconite Workers

Author and Year	Characteristics of Cases	Characteristics of Controls	Study Period	Exposure Categories	Cases	Co	OR	95% CI
Allen et al. (2015b)	1706 lung cancers identified by death certificates and cancer registry data	3381 controls matched on age, two controls selected from risk sets using incidence density sampling	1960−2010 (death certificates) 1988−2010 (cancer registry data)	*By Department* *				
				Mining	NR	NR	0.99	0.97−1.01
				Crushing	NR	NR	0.96	0.88−1.05
				Concentrating	NR	NR	0.99	0.93−1.06
				Pelletizing	NR	NR	1.02	0.97−1.07
				Shop mobile	NR	NR	0.99	0.98−1.01
				Shop stationary	NR	NR	1.01	0.98−1.05
				Office	NR	NR	0.95	0.92−0.99
				*EMP exposure quartiles, EMP/ cm^3^‐years* ^†^				
				Hematite only	738	1532	0.81	0.67−0.98
				0 ‐ <0.1298	NR	NR	1.0	Referent
				0.1298‐<0.4527	NR	NR	1.0	0.79−1.25
				0.4527‐<2.353	NR	NR	0.98	0.77−1.24
				≥2.353	NR	NR	0.82	0.57−1.19
				*Silica exposure quartiles, mg/m* *^3^* *‐years* ^‡^				
				Hematite only	NR	NR	0.81	0.68−0.98
				0 ‐ <0.0373	NR	NR	1.0	Referent
				0.0373‐<0.2064	NR	NR	1.04	0.84−1.29
				0.2064‐<0.5189	NR	NR	0.95	0.74−1.22
				≥0.5189	NR	NR	0.97	0.70−1.35
				*Taconite years by zone* ^§^				
				Zone 1	347	642	1.01	0.97−1.04
				Zone 2	366	618	0.99	0.96−1.02
				Zone 4	327	699	0.99	0.96−1.01
				*EMP/ cm^3^‐years by zone* ^§^				
				Zone 1	347	642	1.00	0.87−1.16
				Zone 2	366	618	0.94	0.85−1.02
				Zone 4	327	699	0.95	0.89−1.01
								
Lambert et al. (2016)	80 mesothelioma cases identified through cancer registry and out‐of‐state death certificates	315 controls (4 controls per case) matched on age and selected from risk sets (incidence density sampling)	1988−2010	*Taconite years* ^¶^	57	184	1.03	1.00‐1.06
				*Taconite years by zone* ^‖^				
				Zone 1	18	74	1.05	1.00−1.11
				Zone 2	31	58	1.06	1.02−1.09
				Zone 4	12	66	0.97	0.92−1.03
				*EMP/ cm^3^‐years by zone* **				
				Zone 1	18	184	1.96	1.15−3.34
				Zone 2	31	92	1.31	1.12−1.54
				Zone 4	12	92	0.88	0.71−1.09
				*EMP/ cm^3^‐years, cumulative* ^††^	57	184	1.10	0.97−1.24
				*High vs. low* ^††^				
				<40 EMP/ cm^3^‐years	17	92	1.0	Referent
				≥40 EMP/ cm^3^‐years	40	92	2.25	1.13−4.50
				*Cumulative exposure tertiles, EMP/ cm^3^‐years* ^††^				
				0 to <0.16	15	61	1.0	Referent
				≥0.16 to <1.15	11	62	0.69	0.28−1.68
				≥1.15	31	61	1.97	0.89−4.32

* Adjusted for years in `unknown' similarly exposed groups (SEGs), hematite, general mine, general plant, general shop, sex, and asbestos.

^†^ Adjusted for hematite exposure, silica exposure, asbestos exposure, and sex.

^‡^ Adjusted for taconite exposure, hematite exposure, asbestos exposure, and sex.

^§^ Adjusted for hematite exposure, silica exposure, asbestos exposure, exposure in other zones, and sex.

^¶^ Adjusted for age and years of employment in hematite.

^‖^ Adjusted for age, employment in hematite mining and employment in other zones.

** Adjusted for age, employment in hematite mining, and potential for commercial asbestos exposure, and exposures in other zones.

^††^ Adjusted for age, employment in hematite mining, and potential for commercial asbestos exposure.

The TWHS investigators reported excess mortality from mesothelioma (SMR 2.77, 95% CI 1.87–3.96, based on 30 mesotheliomas deaths) (Allen et al., 2014), and excess incidence from mesothelioma (SIR 2.4, 95% CI 1.8–3.2, based on 51 mesotheliomas) (Allen et al., 2015a) across the range (Table IV). Lambert et al. (2016) conducted a case‐control study of 80 mesothelioma cases nested within the Mineral Resources Health Assessment Program (MRHAP) miners’ cohort of 68,737 hematite and taconite miners; the underlying study cohort was larger for the mesothelioma case‐control study than for the lung cancer case‐control study and the TWHS cohort mortality and cancer incidence studies. Of the 80 mesothelioma cases, 57 had been employed in taconite mining. When miners across the range were considered as a group, risk of mesothelioma increased by 3% for each year of employment in the taconite industry (relative risk [RR] = 1.03, 95% CI 1.00−1.06) after adjusting for age and years of employment in hematite mining. Risk of mesothelioma increased by 10% for each EMP/cm^3^‐years (RR = 1.10, 95% CI 0.97−1.24) after adjusting for age, employment in hematite mining, and potential for commercial asbestos exposure.

Risk of mesothelioma was associated with duration of taconite employment years for miners located in Zone 2 of the MIR (RR 1.06, 95% CI 1.02−1.09), a zone which included one inactive mine that also mined pits located in Zone 3, but not for taconite miners who worked in Zone 4, the eastern zone where the Northshore mine is located (RR = 0.97, 95% CI 0.92−1.03) (Lambert et al., 2016) (Table IV). Similarly, risk of mesothelioma associated with cumulative EMP exposure was statistically significantly increased for workers in Zone 1 and Zone 2 but not in Zone 4 (Zone 4: RR 0.88, 95% CI 0.71−1.09) (Table IV). Estimated levels of cumulative EMP exposure for cases were lowest in Zone 4, where amphiboles were present in ore (mean of 0.4 EMP/cm^3^‐years) compared to Zones 1 and 2 (mean of 0.5 EMP/cm^3^‐years and mean of 1.4 EMP/cm^3^‐years, respectively), where nonamphibole EMPs predominate.

Because EMPs were analyzed using the NIOSH 7400 method, which does not distinguish amphibole from nonamphibole or asbestiform from nonasbestiform EMPs, the fact that no increased risk of mesothelioma was seen among Northshore workers suggests that exposure to nonasbestiform amphibole EMPs (cleavage fragments) was not responsible for the increased risk of mesothelioma in the cohort. In contrast, risk of mesothelioma was statistically significantly increased in the western end of the range, where EMPs were comprised of crushed ore and phyllosilicates that are neither amphibole EMP nor regulated as asbestiform EMP.

As of 2015, the MDH had reported more than 100 total mesotheliomas in the MRHAP cohort (MDH, 2015); nevertheless, the MDH concluded “*based on available evidence, the elevation in mesothelioma cases in Northeastern Minnesota is most likely an occupational health concern, and does not reflect any increased risk for the broader community*” (MDH, 2015). Since then, Perlman et al. (2018) reported that chest x‐rays of spouses of taconite workers have not shown pleural or parenchymal abnormalities above background. This finding provides supporting evidence of a lack of exposure to EMPs for community members who were not themselves occupationally exposed to asbestos.

### 4.2 Coherence with Existing Toxicological Evidence

To our knowledge, there are no toxicological studies that evaluated a mix of crushed taconite ore containing EMPs or evaluated gangue (waste rock that has no commercial value) minerals found in the western end (zones 1 and 2) of the range (minnesotatite, greenalite, and stilpnomelane). Fibrous ferroactinolite sampled from the iron ore mine in the eastern zone was associated with lung tumors and mesothelioma in rats dosed with 0.5 mg injected via intratracheal instillation over 12 weeks or a single dose of 20 mg via intrapleural injection (Coffin et al., 1982; Cook et al., 1982). Scant information on the source of the sample was provided in these studies although Coffin et al. (1982) reported “test fibers were prepared from loose surface iron‐formation rocks.” The authors, however, did not describe whether the sample was selected because the minerals were typical or atypical of the ore. Ross, Nolan, and Nord (2008) described the fibrous ferroactinolite in place at the mine as localized and contained within readily identified geological features (faults, shear planes, within folds, and dilation cavities), and representing a very small mass of rock in the mine.

Most animal carcinogenicity studies of nonasbestiform amphibole EMPs compared mesothelioma incidence of asbestos and nonasbestos varieties of tremolite (Smith, Hubert, Sobel, & Marquet, 1979; Stanton et al., 1981; Wagner et al., 1982). None of these studies specifically evaluated nonasbestos varieties of cummingtonite‐grunerite. Largely consistent findings were reported in these studies that showed nonasbestiform tremolite was markedly less carcinogenic than tremolite asbestos in rodents dosed via intrapleural injection or intratracheal implantation (Smith et al., 1979; Stanton et al., 1981; Wagner et al., 1982). Stanton et al. (1981) used a gelatin matrix that hardened and held the fibers in contact with the pleura. This method likely prevented particle clearance mechanisms such as macrophage phagocytosis and ciliated cell clearance of the particles from the lungs. Thus, these dosing techniques created the highest effective dose of all the exposure methods used for assessing the potential carcinogenicity of EMPs. This increased the probability that a positive carcinogenic response—if a carcinogenic response is indeed caused by the administered material—would be observed. These exposure techniques subvert the normal physiological defense mechanisms that would operate *in vivo* when materials are administered via the normal inhalation route.

Mossman (2008) reported that *in vitro* studies demonstrate nonasbestiform cleavage fragments are not as strong nor flexible as asbestiform minerals and are inactive in *in vitro* bioassay supporting lower toxicity due to a lack of biopersistence or bioactivity. Toxicological data from *in vivo* and *in vitro* studies collectively provide evidence that nonasbestiform amphibole minerals do not induce mesotheliomas and have limited toxicity with respect to lung tumors.

### 4.3 Supporting Evidence

During 2008–2014, the Natural Resources Research Institute (NRRI) at the University of Minnesota Duluth conducted an environmental study of airborne particulate matter in communities across the Minnesota Iron Range (Monson Geerts et al. 2019). NRRI researchers collected aerosol particulate matter using a Micro‐Orifice Uniform Deposit Impactor (MOUDI) and Total Filter Sampler (TFS) at five community sites (including the Babbitt Municipal Building and the Silver Bay High School), six taconite processing facilities (including Northshore), and three background locations. Monson Geerts et al. (2019) reported a total of 669 EMPs detected in 73 ambient air monitoring samples collected from MIR communities. The EMPs detected included 117 EMPs with AR ≥3:1 that were amphiboles (cummingtonite‐grunerite, actinolite, and tremolite, but not hornblende^1^) or chrysotile. Of these 117 EMPs, only 10 EMPs (1.5% of all EMPs) had length >5 μm in concentrations ranging from 0.003 EMP/cm^3^ to 0.0018 EMP/cm^3^. The 10 EMPs were from community monitoring stations in Virginia, Babbitt, and Silver Bay (Monson Geerts et al., 2019). Only one EMP had an AR ≥15 μm. The average concentrations of countable (length >5 μm and AR≥3:1) and NIOSH “covered” minerals for the community sites, and background sites ranged from nondetected to approximately 0.0003 EMP/cm^3^. Overall, the NRRI study provides a very limited snapshot of sampling events and should be considered less representative of the long‐term ambient air than the Northshore dataset.

Separately, the NRRI sampled lake sediment from two locations in the MIR—one lake located in the eastern zone and one lake located in the western zone—with the purpose of using age‐dating methods to evaluate whether EMPs in sediment originated from atmospheric direct deposition of fugitive dust generated during iron ore mining and related activity. Zanko, Reavie, and Post (2019) analyzed elutriated lake sediment fractions using MDH Method 851 (MDH, 1991b). Zanko et al. (2019) reported a total of 790 EMPs with AR ≥3:1, of which 428 were found in western zone lake sediment and 362 were found in the eastern zone lake sediment. More than 70% of the EMPs were not amphibole and not chrysotile. Only six EMPs had length >5 μm: four were identified as actinolite (three from the eastern zone lake sediment and one from the western lake zone sediment) and two were identified as nonamphibole/nonchrysotile. In addition, seven chrysotile EMPs were found: one in the eastern zone lake sediment and six in the western zone EMPs. All chrysotile EMPs were dated after mining and other industrialized activities began on the MIR. Subsequently, Zanko et al. (2019) concluded that these EMPs originated from anthropogenic sources because chrysotile was not found in local or regional geological sources and was not found in sediments dated before mining activities began.

### 4.4 Integration of Evidence

Characteristics of EMPs known to affect their biological activity (and hazard potential) include physical dimensions, solubility, and biopersistence (NIOSH, 2011). Although certain investigators have argued that health effects associated with shorter mineral EMPs cannot be ruled out as causes or contributors to mesothelioma or lung cancer (Aust, Cook, & Dodson, 2011; Cook et al., 2016; Dodson, Atkinson, & Levin, 2003; Suzuki, Yuen, & Ashley, 2005), there is a general consensus that long and thin amphibole asbestos fibers are more potent for mesothelioma and lung cancer than shorter fibers (Barlow, Grespin, & Best, 2017; Berman & Crump, 2008; Mossman, 2008, 2018; Roggli, 2015; Wylie, Bailey, Kelse, & Lee, 1993, among others). Epidemiological and toxicological evidence indicates that cancer risks associated with shorter asbestos EMPs (length <5 μm) are “very low and could be zero” (Eastern Research Group (ERG) for the ATSDR, 2003) and that fibers of these short lengths are unlikely to cause cancer based on “strong weight of evidence” (ERG for the ATSDR, 2003). Many researchers (Boffetta, Mundt, & Thompson, 2018; Gamble & Gibbs, 2008; Ilgren, 2004; Williams, Dell, Adams, Rose, & Van Orden, 2013) have similarly reviewed evidence regarding nonasbestiform amphibole EMP exposure and concluded that these EMPs do not confer an asbestos‐like risk of cancer. Mandel and Odo (2018) stated that that EMP industrial hygiene monitoring data collected by THWS investigators during 2010–2011 are unlikely to represent past exposure to asbestiform EMP, such as commercial asbestos. In 2017, the Monticello Conference on Elongate Mineral Particles concluded “EMPs with a length less than 5 μm present insignificant risk for EMP‐related cancer” (Weill, 2018).

Residents of Silver Bay are not exposed to concentrations of amphibole EMPs similar to taconite workers. The most compelling explanation for increased risk of mesothelioma in taconite workers is the simplest: these workers were occupationally exposed to commercial asbestos used historically in mining insulation, construction materials, and taconite processing equipment, or asbestos exposure in other occupations. Commercial asbestos was used beginning as early as the late 1940s. This explanation is consistent with the TWHS investigators conclusions as well: *“Given the lack of cancer findings in other studies of non‐asbestiform amphibole exposure, and that no association was observed in the region of the Mesabi Range where non‐asbestiform amphibole minerals are most common, it seems unlikely that non‐asbestiform amphibole exposure would account for the total mesothelioma risk demonstrated in this study”* (Mandel, Alexander, & Ramachandran, 2016, p. 12).

Associations between nonamphibole, nonasbestos EMPs and mesothelioma have not been reported in the literature to our knowledge, with the exception of erionite, a zeolite. Erionite is not reported as present in the MIR (McSwiggen & Morey, 2008; Ross et al., 2008; Zanko, Niles, & Oreskovich, 2008). Amphibole minerals (of any habit) are not found in the ore in the west end of the range, and some of the phyllosilicates that have been hypothesized as a potential culprit are not found in the east. Toxicology and animal data demonstrating carcinogenicity are currently lacking for these phyllosilicates as well. Although chrysotile is a phyllosilicate, its presence on the Iron Range has not been reported (McSwiggen & Morey, 2008; Zanko et al., 2008). Chrysotile is also widely recognized to be less potent than amphibole asbestos for mesothelioma (Berman & Crump, 2008; Hodgson & Darnton, 2000; NIOSH, 2011). Furthermore, lung cancer risks are not increased in taconite workers across the MIR (Allen et al., 2015a, 2015b).

Collectively, the evidence indicates that the Silver Bay community may be exposed to very low concentrations of exposure to ambient amphibole EMPs—specifically cleavage fragments and prismatic crystals, counted as potentially asbestiform using NIOSH EMP 7400 counting rules—over a lifetime. If these EMPs were truly asbestos, the actual risks from such low ambient air concentrations are small and may actually be zero. The Silver Bay EMPs, characterized using TEM, a Modified Elutriator Method (EPA 540‐R‐97‐028), and scanning electron microscopy are described as resembling cleavage fragments (Monson Geerts et al., 2019; Ross et al., 2008). Similarly, Harper et al. (2008) described a population of cleavage fragments, and not asbestiform minerals in air monitoring samples collected from the Northshore facility.

In conclusion, there is a lack of any empirical evidence supporting increased cancer risk from mesothelioma or lung cancer in the Silver Bay community that can be attributed to ambient nonasbestiform amphibole EMPs generated during taconite processing. The existing epidemiological and toxicological data do not support increased risk of cancers from nonasbestiform amphibole EMPs in Northshore workers (Allen et al., 2014, 2015a, 2015b; Lambert et al., 2016). Although cumulative EMP exposure was associated with the prevalence of pleural abnormalities in taconite workers on the MIR, the association was not specific to workers potentially exposed to nonasbestiform amphibole EMPs and the prevalence of pleural abnormalities was not elevated in the spouses of workers (Perlman et al., 2018). The results of particulate matter characterization from independent sampling of ambient air in the MIR communities is consistent with the Silver Bay ambient air monitoring data (Monson Geerts et al., 2019). Sampling of lake sediment conducted by NRRI researchers provides some evidence of past use of commercial asbestos since mining operations began in MIR communities (Zanko et al., 2019). Based on an integration of evidence, an increased risk of mesothelioma and/or lung cancer to residents of the Silver Bay community from potential exposure to cleavage fragments with a length‐to‐width ratio ≥3 μm and length >5 μm emitted during taconite ore processing is vanishingly small, and may be zero.

## Supporting information


**Supplemental Figure 1**. Concentration of amphibole EMPs in all samples, 1990‐2018 (n=1,245).
**Supplemental Figure 2a**. Concentration of amphibole EMPs, 1996‐2018 (n=926).
**Supplemental Figure 2b**. Distribution of lengths for amphibole EMPs, 1996‐2018 (n=6,175).

## Footnotes

1 Hornblende is an amphibole that is not a “covered mineral” under the NIOSH REL or other federal asbestos regulations.
