# Supplementary material for: Integration of Evidence on Community Cancer Risks from Elongate Mineral Particles in Silver Bay, Minnesota
Source: Risk Anal. 2021 Feb 2;41(9):1674–92. doi: 10.1111/risa.13673 (PMC8596417; doi:10.1111/risa.13673)
Supplement: Supplementary file 1 — Supplemental Figure 1. Concentration of amphibole EMPs in all samples, 1990‐2018 (n=1,245). Supplemental Figure 2a. Concentration of amphibole EMPs, 1996‐2018 (n=926). Supplemental Figure 2b. Distribution of lengths for amphibole EMPs, 1996‐2018 (n=6,175). [file RISA-41-1674-s001.docx]

*Blue dots represent the concentration of each sample. Orange dots represent the rolling mean.

Supplemental Figure 1. Concentration of amphibole EMPs in all samples, 1990-2018 (n=1,245)

*Blue dots represent the concentration of each sample. Orange dots represent the rolling mean.

†24 fibers with amphibole concentrations greater than 0.02 f/cc are not visible.

Supplemental Figure 2a. Concentration of amphibole EMPs, 1996-2018 (n=926)

Supplemental Figure 2b. Distribution of lengths for amphibole EMPs, 1996-2018 (n=6,175)
